# Neonatal Purpura Fulminans in a Patient With Arterial Thrombosis and Congenital Renal Agenesis

**DOI:** 10.7759/cureus.52503

**Published:** 2024-01-18

**Authors:** Mawada M Abbaker, Srujan Edupuganti, Sheimaa T Elmukashfi, Taha Ahmed E Elsheikh

**Affiliations:** 1 Pediatrics, Hurley Medical Center, Flint, USA; 2 Internal Medicine/Pediatrics, Hurley Medical Center, Flint, USA; 3 Paediatrics, Gaafar Ibn Auf Teritiary Hospital, Khartoum, SDN; 4 Community Medicine, University of Khartoum, Khartoum, SDN

**Keywords:** sudan, high index suspicion, protein s deficiency, protein c deficiency, neonatal purpura fulminans

## Abstract

Neonatal purpura fulminans (PF) is an uncommon skin disorder characterized by acute disseminated intravascular coagulation, tissue necrosis, and small vessel thrombosis. Here, we present a case of a seven-day-old male who was admitted to the Neonatal Intensive Care (NICU) Unit at Gaafar Ibn Auf Tertiary Hospital, in January 2023. He presented with black bullous lesions on the plantar surface of the left foot, deep bluish discoloration over the right buttock and right lower abdomen, and gangrenous changes in the right foot with clear demarcation. Birth history was not significant other than mild pallor and icterus. His blood workup was consistent with severe anemia, thrombocytopenia, elevated prothrombin time, and partial thromboplastin time with decreased protein C and S levels; blood culture yielded no growth. A Doppler ultrasound scan of lower extremities confirmed distal occlusion of the right dorsalis pedis artery. The abdominal ultrasound revealed a free left renal bed and left-sided renal agenesis. We came to a diagnosis of neonatal PF and started administering blood and fresh frozen plasma and subcutaneous heparin injections, but unfortunately, the patient eventually passed away. Hence, we decided to report this case to emphasize the significance of the clinical picture in assisting with early diagnosis, despite limited access to genetic testing. We also want to highlight the importance of a “high index of suspicion” that might be mandatory for better outcomes.

## Introduction

Neonatal purpura fulminans (PF) is a rare fatal disorder that presents commonly with signs of disseminated intravascular coagulation (DIC), small vessel thrombosis, and tissue necrosis. This condition could be congenital or acquired. The congenital cause results from homozygous or double heterozygous protein C (PC) and protein S (PS) deficiency, which is inherited as autosomal dominant [1]. Those vitamin K-dependent glycoproteins are natural anticoagulants. PC is activated by thrombin on the endothelial surface; thrombomodulin and endothelial PC receptors are involved in this process. Activated PC requires calcium, phospholipids, and PS to deactivate factor V (FVa) and factor VIII (FVIIIa) by cleaving arginine residue at three specific sites [2]. Infection is the most commonly reported reason for acquired PF and the most commonly reported organisms are *Streptococcus pneumoniae*, *Meningococcus*, *Escherichia coli*, *Varicella*, *Klebsiella oxytoca*, and *Citrobacter* [3]. Hereditary deficiency of PC, PS, and antithrombin-III is described as neonatal purpura fulminans. Idiopathic PF is a post-infectious autoimmune disorder that starts as a febrile illness and eventually turns out to be a rapidly progressive purpura [4].

## Case presentation

**Figure 1:**
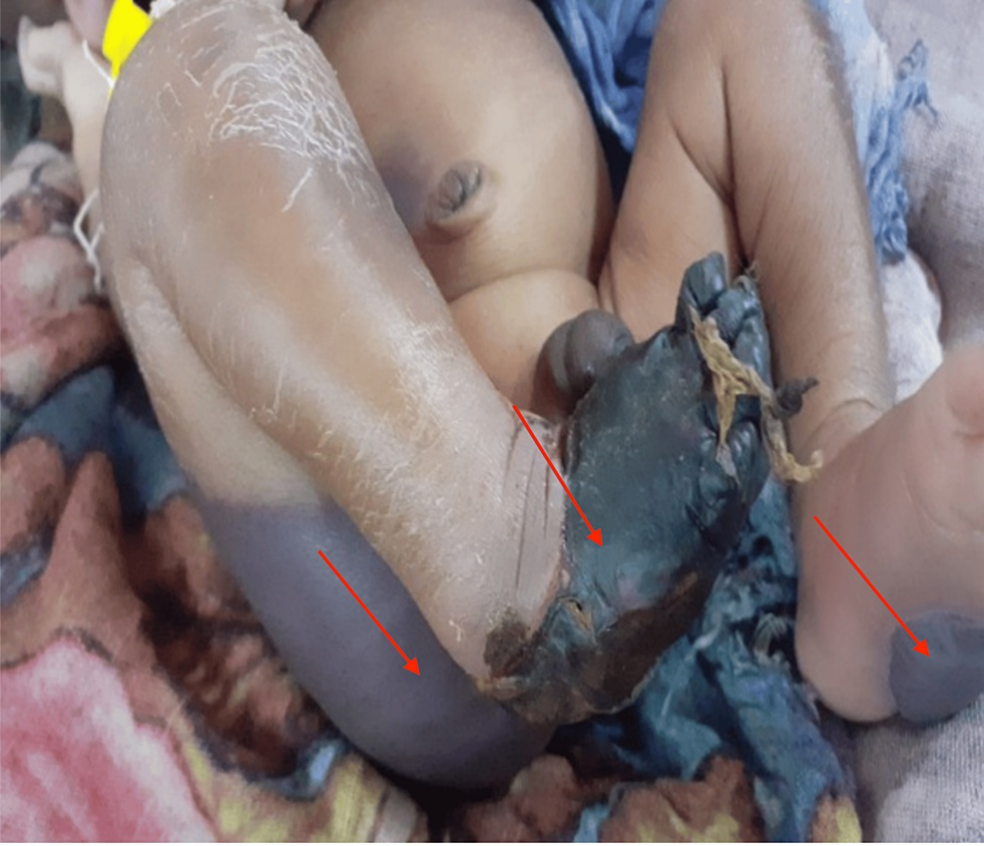
Violaceous purpuric macule over the right buttock, gangrenous right foot and black bullae over the left sole

A seven-day-old male born via an emergency cesarean section due to twin pregnancy and breech presentation (the other twin is a female, alive and well) was referred to our neonatal intensive care unit (NICU) in January 2023 due to black bullous lesions on the plantar surface of the left foot and deep bluish discoloration over the right buttock and right lower abdomen. In addition, the right foot was gangrenous with clear demarcation (Figure 1). Those lesions were developed on the second day. Also, the right foot gangrene started as a black bullous lesion that then ruptured. The mother was 23 years old with regular antenatal care and had a medical history of two first-trimester miscarriages. The parents were first-degree relatives and had two children. The infant weighed 2100 g at birth and had no apparent congenital anomalies. He was hemodynamically stable but had mild icterus and pallor. Systemic examination was unremarkable apart from the skin lesions. Neonatal reflexes were positive. He was active, and feeding was normal. His blood workup was significant for severe anemia, thrombocytopenia, indirect hyperbilirubinemia, elevated prothrombin time (PT) and partial thromboplastin time (PTT), elevated C reactive protein (CRP), and decreased levels of PC and PS (Table 1). A Doppler ultrasound scan of the lower extremities confirmed distal occlusion of the right dorsalis pedis artery. The abdominal ultrasound revealed a free left renal bed and left-sided renal agenesis.

**Table 1:** Blood workup at admission INR, international normalized ratio

Blood test	Results (normal value)
Hemoglobin	4.9 g/dl (13.5-17.5)
Hematocrit	14% (45-61)
White cell count	18,600/cmm (9000-30,000)
Neutrophils	80% (60-70)
Lymphocytes	15% (20-30)
Platelets	45,000/cmm (150,000-350,000)
Total bilirubin	6.67 mg/dl (0.2-1.3)
Indirect bilirubin	5.72 mg/dl (0.2-0.8)
Prothrombin time	32.3 seconds (11-13.5)
INR	2.62 (1.1)
Partial thromboplastin time	61.2 seconds (21-35)
C-reactive protein	49.57 mg/dl (0.3-1.0)
Protein C	41% IU (65-135)
Protein S	59% IU (60-150)

**Figure 2:**
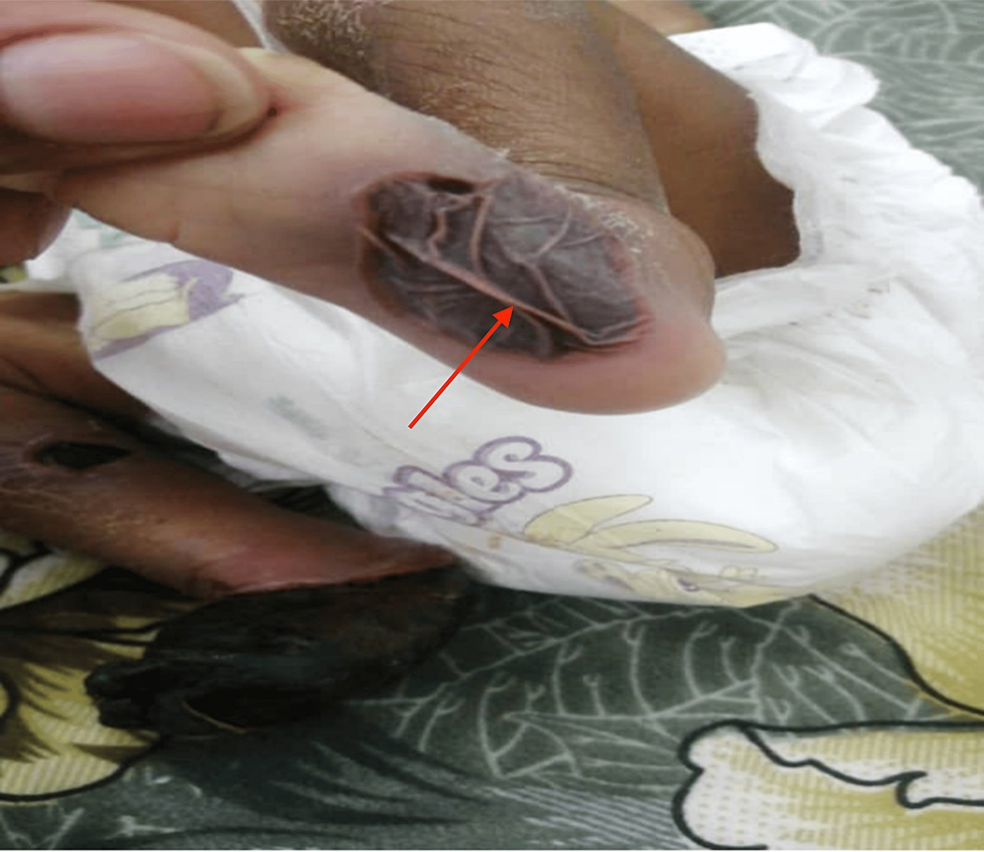
Ruptured soft bullae over the left foot

**Figure 3:**
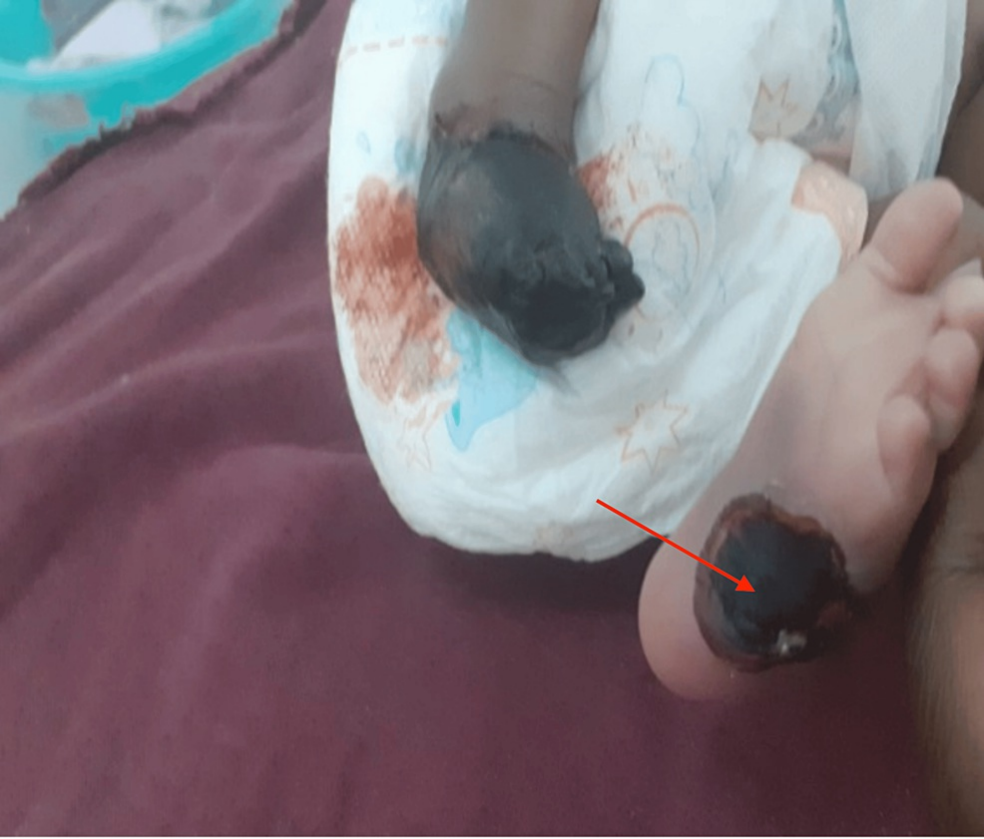
Left sole ulcer covered with eschar

**Figure 4:**
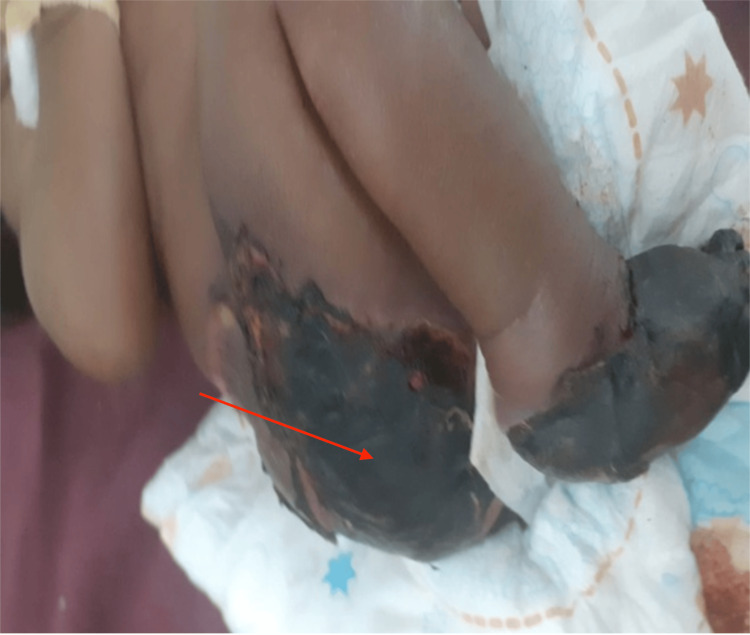
Hemorrhagic skin necrosis with black eschar formation

The infant received blood and fresh frozen plasma transfusions during the hospital course and was commenced on antibiotics. Blood culture yielded no growth. Hematology consultation was done and low molecular weight heparin was added as well. A repeat blood workup after 48 hours revealed improved hemoglobin, platelet count, PT, and PTT levels (Table 2). The left foot bullous lesion ruptured leaving an ulcer (Figures 2, 3). The violaceous purpuric macule over the right buttock got necrotic with black eschar formation and superficial skin sloughing that then got infected (Figure 4). The infant was referred to the surgical team for debridement and right foot amputation. Unfortunately, the patient had an episode of hemorrhagic necrosis, mesenteric ischemia, and ended up with disseminated intravascular coagulation, eventually passing away.

**Table 2:** Blood workup after 48 hours of admission INR, international normalized ratio

Blood test	Results
Hemoglobin	11.1 g/dl
Hematocrit	33.4%
White cell count	15,500/cmm
Platelets	386,000/cmm
Prothrombin time	16.1 seconds
INR	1.26
Partial thromboplastin time	38.9 seconds

## Discussion

Chakravarty et al. also discussed a case of arterial thrombosis involving the distal aorta, where the parents were first-degree relatives with a history of recurrent fetal losses. That infant PC level was less than 10% and no genetic test was done [5]. In another case, an infant born to first-degree relatives presented with bilateral adrenal hemorrhage and neonatal PF, and had decreased PC levels (24%); the parents had moderately decreased PC levels (46%, 52%) although they were not symptomatic [6]. A study done in Japan showed that most patients with neonatal onset PC deficiency had a level of less than 10%; however, late-onset patients had a median PC level of 31% (range 19%-52%). Those infants with double mutations of the PC gene (PROC) develop PF and stroke. They rarely escape infancy and childhood without thrombosis. Individuals heterozygous for PROC are predisposed to thrombosis when infected or distressed [7]. A case series of 12 Chinese infants revealed that all the cases with neonatal PF, except one, had a protein C level of less than 10% [8]. Heterozygous protein C deficiency is associated with less severe symptoms and is unlikely to present with arterial thrombosis [9]. The hereditary severe protein C deficiency is very rare with a frequency of 1 in 40,000-250,000; the homozygous variant occurs in 500,000-750,000 with an equal distribution of males and females [10]​. Skin involvement is the earliest manifestation of this serious condition. The skin findings of PF have a characteristic appearance and evolution that aid in diagnosis [4]. Purpura fulminans from homozygous protein C or protein S deficiencies occurs in two phases. The first phase is when the reversible lesions develop and grow. The stage could be halted and reversed with the administration of protein C or protein S [11]. The second phase is irreversible in which the lesions get necrotic whether or not treated with protein C​ [12]. The differentiation between acquired and congenital PC deficiency is essential because the former usually follows an infection and is manageable with fresh frozen plasma in resource-limited settings, and heparin or warfarin need not be commenced ​[13]. Genetic/molecular studies for both the patient and parents are important in terms of prognosis and genetic counseling. Even with a typical presentation, the functional assays of PC could be negative, as seen in a four-year-old female from Chicago with purpura fulminans and negative assays for thrombophilia who came positive for compound heterozygous protein C deficiency when tested using whole exome sequencing [9]. A preterm infant in Germany was successfully treated with protein C concentrate up to adolescence. Another promising treatment option is liver transplantation since protein C is produced in the liver. A Korean infant showed good overall development after liver transplantation with no complications up to eight years post-transplantation [14].

This infant's CRP was high, and his blood culture was negative that makes an acquired protein C/S deficiency unlikely. No genetic analysis was done for this infant; however, the severity of his condition advocates for homozygous or compound heterozygous PROC mutation. Heterozygous protein C deficiency is associated with less severe symptoms and is unlikely to present with arterial thrombosis. This infant had confirmed dorsalis pedis thrombus that led to right foot gangrene and ended with amputation​. Moreover, the mother's history of two first-trimester miscarriages and the infant's absent left kidney support an in utero thrombotic event. Also, being a child of a consanguineous marriage further indicates the underlying role of genetic factors. Here, the parents had no symptoms, and we did not check their protein C level. The infant had a PC level of 41%, and severe symptoms developed very early within hours after birth, which contradict other reported cases. However, the clinical picture of bullous lesions getting ruptured and turning necrotic was similar to many internationally reported cases. Protein C concentrate to be given to our patient to halt the progress into necrosis was not available at our hospital, which would have helped in reversing the condition if given in the early stages of the disease [13].

## Conclusions

Despite lack of access to genetic testing in resource-limited settings, the clinical picture plays a significant role in the early diagnosis of neonatal purpura fulminans. Given the better outcomes seen with early-stage diagnosis and treatment, having a high index of suspicion should be mandated.
